# Feasibility and Efficacy of Delivering Cognitive Behavioral Therapy Through an Online Psychotherapy Tool for Depression: Protocol for a Randomized Controlled Trial

**DOI:** 10.2196/27489

**Published:** 2021-06-16

**Authors:** Nazanin Alavi, Callum Stephenson, Megan Yang, Anchan Kumar, Yijia Shao, Shadé Miller, Caitlin S Yee, Anthi Stefatos, Maedeh Gholamzadehmir, Zara Abbaspour, Jasleen Jagayat, Amirhossein Shirazi, Mohsen Omrani, Archana Patel, Charmy Patel, Dianne Groll

**Affiliations:** 1 Department of Psychiatry Queen's University Kingston, ON Canada; 2 Centre for Neuroscience Studies Queen's University Kingston, ON Canada; 3 Department of Psychology University of Toronto Toronto, ON Canada; 4 OPTT Inc Digital Media Zone Ryerson University Toronto, ON Canada

**Keywords:** mental health, depression, psychotherapy, cognitive behavioral therapy, online, internet, electronic, virtual, mental health care

## Abstract

**Background:**

Major depressive disorder (MDD) is a prevalent and debilitating mental health disorder. Among different therapeutic approaches (eg, medication and psychotherapy), psychotherapy in the form of cognitive behavioral therapy (CBT) is considered the gold standard treatment for MDD. However, although efficacious, CBT is not readily accessible to many patients in need because of hurdles such as stigma, long wait times, high cost, the large time commitment for health care providers, and cultural or geographic barriers. Electronically delivered cognitive behavioral therapy (e-CBT) can effectively address many of these accessibility barriers.

**Objective:**

This study aims to investigate the efficacy and feasibility of implementing an e-CBT program compared with in-person treatment for MDD. It is hypothesized that the e-CBT program will offer results comparable with those of the in-person treatment program, regarding symptom reduction and quality of life improvement.

**Methods:**

This nonrandomized controlled trial intervention will provide e-CBT for MDD through the Online Psychotherapy Tool, a secure, cloud-based, digital mental health platform. Participants (aged 18-65 years) will be offered 12 weekly sessions of an e-CBT program tailored to MDD to address their depressive symptoms. Participants (n=55) will complete predesigned modules and homework assignments while receiving personalized feedback and interacting with a therapist through the platform. Using clinically validated symptomology questionnaires, the efficacy of the e-CBT program will be compared with that of a group (n=55) receiving in-person CBT. Questionnaires will be completed at baseline, at week 6 and week 12, and at a 6-month follow-up. Focus groups will be conducted to investigate personal, cultural, and social factors impacting the accessibility and feasibility of implementing a web-based psychotherapy tool from a patient and care provider perspective. Inclusion criteria include diagnosis of MDD, competence to consent to participate, ability to speak and read English, and consistent and reliable access to the internet. Exclusion criteria include active psychosis, acute mania, severe alcohol or substance use disorder, and active suicidal or homicidal ideation.

**Results:**

Ethics approval was obtained in January 2019, and recruitment of participants began in June 2019. Recruitment has been conducted via social media, web-based communities, and physician referrals. To date, 52 participants have been recruited to the e-CBT group, and 48 patients have been recruited to the in-person CBT group. Data collection is expected to be completed by March 2021, and analyses are expected to be completed by June 2021, as linear regression (for continuous outcomes) and binomial regression analysis (for categorical outcomes) are still being conducted.

**Conclusions:**

The results of this study can provide valuable information for the development of more accessible and scalable mental health interventions with increased care capacity for MDD, without sacrificing the quality of care.

**Trial Registration:**

ClinicalTrials.gov NCT04478058; http://clinicaltrials.gov/ct2/show/NCT04478058

**International Registered Report Identifier (IRRID):**

DERR1-10.2196/27489

## Introduction

### Background and Rationale

More than 264 million people globally have depression, with major depressive disorder (MDD) being one of the most prevalent mental health disorders [1]. In addition, depression is the largest contributor to global disability and is associated with an estimated economic cost of more than US $2.5 trillion globally [2-6]. Given the large prevalence and the debilitating nature of this mental disorder, discovering effective and accessible treatments for depression is essential and urgently needed.

There are different therapeutic approaches for treating MDD, from medication to psychotherapy. Although pharmacotherapy has been widely investigated and various medications are used to treat MDD, approximately one half of primary care patients only respond partially to medication, with residual symptoms persisting in most cases [7]. Furthermore, many patients using medications for MDD experience adverse effects (eg, weight change, sexual side effects, and sleep problems) or develop addiction or dependency on these medications [8]. These challenges discourage many patients from taking their medications and render this therapeutic path moot as a first-line treatment for MDD.

The current frontline treatment for MDD is in-person psychotherapy, more specifically, cognitive behavioral therapy (CBT) [9-11]. This traditional form of CBT uses a structured format to focus on altering cognitive processing based on the theory that maladaptive behaviors affect an individual’s perceptions, emotions, and thoughts [12]. By altering these maladaptive behaviors, a patient’s thoughts and beliefs can be positively impacted, allowing for a cyclical improvement in cognition and behavior. CBT allows patients to learn the necessary skills to better cope with situations in their lives by better interpreting their thoughts, thereby influencing their emotions. By positively influencing thoughts, mood, behavior, or physical reactions, a positive effect is seen in other areas of one’s life. Through CBT programming, patients can learn skills such as breathing techniques, thought records, activity scheduling, and goal setting, which can give them the tools they need to better control their emotions.

Although CBT is efficacious for treating mood and anxiety disorders, the barriers associated with this treatment make it extremely inaccessible. An effective round of CBT usually requires a large time commitment (ie, 12-15 weekly sessions), making CBT a costly solution for patients. In addition, given the time commitment from the clinicians, there are often extremely long wait times for individuals to receive treatment. Additional barriers to CBT include cultural and language barriers, especially within more culturally diverse countries, geographic isolation in rural areas, and social isolation, particularly in the context of the COVID-19 pandemic. Public stigma, high financial costs, lack of after-hour resources, and confidentiality concerns are also common concerns associated with receiving CBT [13,14]. Therefore, new innovative solutions are needed to make this effective and safe treatment option more accessible to patients in need without sacrificing the quality of care.

Electronically delivered cognitive behavioral therapy (e-CBT) presents itself as a remedy to these barriers. Owing to the structured nature of CBT, it can be effectively delivered remotely in the form of e-CBT. e-CBT offers results comparable with those of the in-person CBT, while increasing treatment adherence and yielding high treatment satisfaction [15,16]. Recent findings have supported the notion of implementing e-CBT for MDD, showing significant improvements in symptoms in various settings [17-19]. The use and accessibility of the internet have continued to grow globally, with more than 40% of the population having access to the internet and more than 2.5 billion individuals currently using the internet [20,21]. Given the increased accessibility to and use of web-based resources, internet-based delivery of mental health treatments can make help accessible and available at any time or place, helping underserved populations, geographically isolated individuals, and people looking for treatment outside of their work hours. In addition, delivering mental health care on the web provides a secure and private environment that could lower the stigma of receiving care, which is particularly useful for patients with MDD who usually show low help-seeking behaviors [22-24]. Furthermore, receiving care on the web allows patients to save time or financial burdens commuting to weekly appointments.

Several forms of e-CBT have been used, including self-help (unguided and self-directed), guided self-help (clinicians provide limited support), and *live* psychotherapy (clinician has the same role as in-person CBT through video conferencing). A meta-analysis [25] revealed that although self-help e-CBT can have varying degrees of efficacy, therapist engagement increases treatment efficacy up to 4 times [26,27]. Therefore, a supervised form of e-CBT is required. Although this supervision can be achieved in *live* psychotherapy through videoconference, the time commitment for clinicians is identical to, if not greater than, that needed for in-person CBT [11]. From this, we can see that an asynchronous form of e-CBT delivery must be used where therapist supervision can be achieved without the strenuous time commitment of in-person CBT. Asynchronous e-CBT has been delivered in previous research; however, these studies used email as the primary method of communication between therapists and patients [28,29]. Although this offers the benefits of therapist engagement, email is an insecure and unsustainable form of patient-clinician communication [30]. This can be achieved by using predesigned and clinically validated therapy content coupled with asynchronous therapist interaction through a secure platform. In doing so, a high standard of care can be achieved without repeating general concepts to all patients, thus saving time.

This study aims to evaluate the efficacy and feasibility of delivering a supervised e-CBT program with streamlined processes for delivering care. By using predesigned therapeutic content, clinicians can skip repeating general concepts across multiple patients and focus on personalizing the treatment. This practice can significantly lower the time commitment from the clinical staff, which lowers the cost of care and increases the capacity, thereby shortening the wait time. Moreover, by validating the therapeutic content and clinical process in this clinical trial, we ensure that a high quality of care is delivered on a routine basis. The predesigned content will be delivered through a secure web-based platform, the Online Psychotherapy Tool (OPTT; OPTT Inc), designed by the lead author. The platform allows clinicians to schedule their interactions and activities with patients, track patient participation, and access analytical tools to improve the quality of care. Clinicians can personalize treatment plans for each patient, similar to *live* psychotherapy, without the time commitment needed for live and face-to-face treatment. By delivering e-CBT for MDD through this web-based platform, the aim is to provide patients with more accessible, affordable, and efficacious treatment options for their problems.

### Objectives

This study aims to implement an e-CBT program to improve symptoms in patients with MDD. This study also aims to deliver this care program through a secure web-based platform structured as a web-based mental health clinic. This e-CBT program will be delivered using a digital care plan (ie, therapeutic content, feedback templates, care procedures, and instructions) specifically designed to address problems faced by patients with MDD. Using qualitative focus groups to collect personal, social, and cultural information from patients and health care providers, the accessibility and feasibility of the program can be better understood.

## Methods

### Study Design

For this study, an open-label, nonrandomized controlled trial study design will be used to compare the efficacy and feasibility of the web-based program with the in-person treatment option in a controlled manner. Blinding will not be possible, as patients will be aware of whether their treatment is occurring in person or remotely. Qualitative focus groups will be conducted with patients to gather personal, social, and cultural factors. These focus groups will be conducted to allow for a natural and open conversation on how participant factors can impact the treatment experience with the program. Additional focus groups will be conducted with therapists to gather information regarding the feasibility of implementing a web-based psychotherapy tool. This will be done to better understand how web-based treatment compares with in-person in terms of usability, time efficiency, and perceived connectedness to patients. These focus groups will occur posttreatment.

Quantitative analyses of e-CBT treatment efficacy will be conducted using standardized and clinically validated symptomology questionnaires. This data collection will occur at baseline, at week 6 and week 12 (posttreatment), and at a 6-month follow-up. In addition, a qualitative analysis of focus group interviews will be conducted with theme extraction. We hypothesize that the e-CBT program will offer results comparable with those of the in-person treatment program regarding symptom reduction and quality of life improvement. On the basis of this design, both the efficacy and feasibility of the e-CBT program can be evaluated and compared with in-person treatment for MDD. All study procedures have been approved by the Queen’s University Health Sciences and Affiliated Teaching Hospitals Research Ethics Board (HSREB; file number 6020045).

### Participants

Patients (N=110; 55 e-CBT participants and 55 in-person CBT participants) aged between 18 and 65 years will be recruited from Queen’s University from the outpatient psychiatry clinic at both the Kingston Health Sciences Center sites (Hotel Dieu Hospital and Kingston General Hospital) as well as from Providence Care Hospital located in Kingston, Ontario, Canada. Additionally, family doctors, physicians, clinicians, and self-referrals will be accepted. Once informed consent is provided, a psychiatrist from the research team will evaluate participants through a secure video appointment to make or confirm a diagnosis of MDD using the Diagnostic and Statistical Manual of Mental Disorders, 5th edition [31].

The inclusion criteria for the study are as follows: aged at least 18 years at the start of the study, diagnosed with MDD according to Diagnostic and Statistical Manual of Mental Disorders, 5th edition by an attending psychiatrist from the research team, competence to consent to participate, ability to speak and read English, and consistent and reliable access to the internet. Exclusion criteria are as follows: active psychosis, acute mania, severe alcohol or substance use disorder, and/or active suicidal or homicidal ideation. If a participant is receiving another form of psychotherapy, they will also be excluded from the study to avoid any confounding effect on the efficacy of this e-CBT program. Participants are eligible for the study if they are receiving pharmacotherapy. However, they must be receiving the same drug and dosage for the duration of the study. If eligible for the study, participants will choose between the e-CBT program (n=55) or the in-person CBT offered individually through this program at Hotel Dieu Hospital (n=55).

During the informed consent process, all participants will be explained that this program is not a crisis resource and that they will not always have access to their therapist. In the case of an emergency, participants will be directed to the proper resources (eg, emergency department or crisis lines), and this event will be reported to the principal investigator of the study. The reasoning behind having a nonrandomized design and allowing patients to choose their treatment is to mimic a real-world setting in which patients have autonomy in their treatment decisions. We recognize that this could be a limitation of this study. We plan to conduct a randomized controlled trial in the future.

### Procedures

The e-CBT care plan consists of 12 weekly sessions of approximately 30 slides and interactive content delivered through OPTT (refer to Multimedia Appendix 1 for the sample module). The e-CBT module content mirrors in-person CBT content, including different weekly topics, general information, skill overviews, and homework. The efficacy of the therapeutic content has previously been tested through email administration and has been shown to reduce depressive symptoms significantly [32]. Participants are instructed to go through the content and complete homework at the end of the session, which helps them practice the skills they learned through that session. Homework is submitted through OPTT and reviewed by the therapist assigned to the participant, who will provide personalized feedback within 3 days of submission. Therapists have access to predesigned session-specific feedback templates to use as a basic structure to write their feedback. By doing so, the time needed to respond to each patient is reduced, and therefore, the number of patients each therapist can handle increases. At the same time, by using a structured format in responding to patients, more standardized quality of care can be ensured. The general structure of the feedback template is as follows: validating the participant’s time and effort, reviewing the event they have used in their homework, summarizing the previous module’s content, and discussing the participant’s homework submission and how they could improve it. In addition to these points, the feedback will emphasize specific content from the participant’s submission, reassuring them that their therapist is reading and understanding their challenges. All feedback submissions are finished with a personalized signature from the therapist, helping to develop a rapport between the therapist and the participant. On average, developing this feedback takes a therapist 15 to 20 minutes per patient. In addition to the weekly feedback, participants have the option to message their therapist through the platform throughout the week regarding any questions or concerns they may have. All technical issues are handled directly by OPTT’s technical support team.

Participants in the in-person CBT group will attend weekly sessions at Hotel Dieu Hospital (12 weeks), where they will receive standardized individual (one-on-one) CBT for MDD from a trained therapist. All content covered and skills taught will mirror the e-CBT program. Similar to the e-CBT group, participants will be assigned weekly homework assignments that they will complete during the week and hand over at the start of their next session. At this time, participants will receive personalized feedback from their therapist for their previous week’s homework.

At the end of the study, a few participants and health care providers (ie, 8 participants and 2 providers) will be recruited for focus groups. The focus group prompts will pertain to experience and expectations of service during the study and how they think the service could be improved.

### Web-Based Module Content

Both e-CBT modules and in-person CBT sessions are designed to instill constructive and balanced coping strategies in the participants. During the program, we focus on essential thinking and behavioral skills to help patients become more engaged in day-to-day activities. The sessions focus on the connection between thoughts, behaviors, emotions, physical reactions, and the environment. In addition, we will evaluate negative beliefs and thought processes and their relationships with depression. Our goal is to adjust negative thinking so that participants can think about and adapt to the things that are happening to them. This allows them to adjust the way they behave and think about their problems in a way that is not as negative and replaces those thoughts and behaviors with potentially more realistic and productive ones. The 12 e-CBT sessions will follow the themes listed below:

1. *What is depression?* Provides expectations for the course and introduces the concepts of CBT and depression.
2. *5-part model:* Introduces the 5-part model (how situations, thoughts, feelings, physical reactions, and behaviors are connected and how they interact with each other).
3. *Situation, thoughts, feelings, physical reactions, and behaviors:* Provides further detailed exploration of the 5-part model and how changes in an area affect the other parts.
4. *Rating feelings and thought records:* Highlights the first 3 columns of a thought record (a tool used to help understand the connection between feelings, behaviors, and thoughts), which are situation, feelings, and automatic thoughts associated with the situation.
5. *Automatic thoughts:* Explains the role of automatic thoughts and how they influence feelings. Provides ways to identify thoughts and specifically identify the most dominant thoughts in stressful situations.
6. *Evidence:* Focusing on columns 4 and 5 of the thought record, which are designed to help gather evidence that supports or does not support the identified automatic thought.
7. *Alternative and balanced thinking:* Focus on the final 2 columns of the thought record that reflect on the evidence columns to help find alternative and more balanced views of the situation. The final column has the patient rerate their feelings based on the completion of the thought record.
8. *Experiments:* Explains the importance of conducting experiments to believe the alternative and balanced thoughts from the thought record to initiate changes in negative thinking patterns.
9. *Action plans:* Centered around using the thought record to identify a problem that needs to be solved and providing a framework for creating a plan for solving the problem.
10. *Strategies for stressful situations:* Overview of helpful strategies that can be used in stressful situations, including distraction activities and helpful breathing techniques.
11. *Activity scheduling:* Explains activity records and how using one can inform the patient on their mood changes and help reinforce scheduling positive activities into their day.
12. *Review:* Summarizes the main concepts of CBT and the tools and skills that the patient should continue to practice beyond completion of the final session.

### Training

All therapists are research assistants hired by the principal investigator. All therapists undergo training in psychotherapy and receive additional training from a psychiatrist from the research team before any interaction with participants. During this training, therapists complete feedback on practice homework, which is reviewed by a psychiatrist from the research team to ensure adequate quality of work. All therapists are supervised by the lead psychiatrist, who is an expert in electronically delivered psychotherapy [23,24]. Feedback is always reviewed by the lead psychiatrist, before submission to the participants.

### Outcome Evaluation

The primary outcomes that will be measured include changes in symptoms of depression based on the following clinically validated symptomology questionnaires: Patient Health Questionnaire-9 (PHQ-9) and the Quick Inventory of Depressive Symptomatology Questionnaire [33,34]. An additional measurement of quality of life change will be made based on the Quality of Life and Enjoyment Questionnaire [35]. All questionnaires were shown to be reliable and highly valid. Questionnaires will be collected directly through OPTT before treatment (baseline), after session 6, after the final session (week 12), and after a 6-month follow-up. Participants will not be offered incentives to increase the completion of the questionnaires. OPTT will collect use statistics (ie, log-ins per day and amount of time spent logged in) to help better understand the relationship between engagement and treatment outcomes.

Through the focus groups, health care providers will be asked about the feasibility of providing the electronic-delivered psychotherapy program and how it compares with in-person psychotherapy delivery. They will be asked to make their judgments based on variables such as time commitment, connectedness to patients, and benefits and drawbacks. From the focus group interviews with the participants, personal, social, and cultural factors (eg, gender, sexuality, background, supportive resources, and structural or social barriers) will be extracted using an interpretive phenomenological analysis approach. In addition, any adverse events and adherence to the intervention will be reported.

### Ethics and Data Privacy

All procedures have been approved by the Queen’s University HSREB. For privacy purposes, participants are only identifiable by an ID number on the platform, and hard copies of the consent forms with participants’ identities are stored securely on site and will be destroyed 5 years after study completion. Participants’ data are only accessible by the care providers directly assigned to that participant, and only anonymized data are provided to the analysis team members. Participants have the option to withdraw from the study at any point and request for their data to be removed from the analysis. However, as the collected data are considered a medical record, it will not be permanently deleted for 10 years after treatment.

The web-based platform used for the study (OPTT) is Health Insurance Portability and Accountability Act, Personal Information Protection and Electronic Documents Act, and Service Organization Control-2 compliant. In addition, all servers and databases are hosted in the Amazon Web Service Canada cloud infrastructure, which is managed by Medstack to ensure that all provincial and federal privacy and security regulations are met. OPTT does not collect any identifiable personal information or internet protocol addresses for privacy purposes. OPTT only collects anonymized metadata to improve its service quality and provide advanced analytics to the clinician team. OPTT encrypts all data, and no employees have direct access to participants’ data. All encrypted backups are kept in the S3 storage that is dedicated to Queen’s University, located in Kingston, Ontario, Canada.

### Data Analysis

This is a confirmatory study with previously established objectives and analyses. Initially, all data are examined for missing, nonsensical, and outlying variables. Missing data are treated as missing and not imputed (ie, will be analyzed on a per-protocol basis). The participants in this study were intentionally oversampled to account for dropouts or withdrawals. On the basis of previous research, an anticipated dropout rate of up to 30% was factored in. Using the PHQ-9 as the primary outcome, a 30% change is considered clinically significant. Therefore, a sample size of 55 participants in each arm of the study would be sufficient for detecting significant results with *P*=.05 and a power of 0.95. Data collection occurs at baseline (preintervention), in the middle of the study (week 6), immediately after the intervention (week 12), and at a 6-month follow-up. Using Mann-Whitney *U* tests, demographic information can be compared between participants who complete the program and those who withdraw prematurely in the hope of identifying possible differences between the two. Moreover, an intention-to-treat analysis will be conducted to evaluate the clinical effects of treatment on participants who withdraw prematurely. Linear regression analysis (for continuous outcomes) and binomial regression analysis (for categorical outcomes) will be used to identify variables associated with the outcome measures (PHQ-9, Quick Inventory of Depressive Symptomatology Questionnaire, and Quality of Life and Enjoyment Questionnaire). This will occur over the 4 measurement time points while controlling for demographic variables, including age and gender. In addition, a comparative analysis between both groups’ questionnaire scores will be conducted at all data collection time points using group and paired two-tailed *t* tests.

Other quantitative measures for the e-CBT group will be gathered by extrapolating recorded information directly through the OPTT platform (eg, the number of log-ins per day and the amount of time spent logged in). Qualitative measurement analyses will be conducted to inquire about the role of personal, social, and cultural factors in enabling or constraining the use of e-CBT. The findings will identify factors related to the utility, feasibility, and accessibility of e-CBT from the perspectives of users and providers. Interpretive qualitative methods are ideal for gathering in-depth descriptions of user experience and meaning.

## Results

This study received ethics approval from the Queen’s University HSREB in January 2019, and the recruitment of participants began in June 2019. Participant recruitment has been conducted through social media advertisements, physical advertisements, and physician referrals. To date, 52 participants have been recruited in the e-CBT arm, and 48 participants have been recruited in the in-person CBT arm. Data collection is expected to be concluded by the end of March 2021, and data analyses are expected to be completed by June 2021.

## Discussion

### Comparison With Prior Work

MDD is a debilitating mental illness. Although treatment options are available, these modalities are either not efficacious for patients, inaccessible, or not scalable to a large population. Therefore, an innovative treatment that is efficacious and accessible while having an increased care capacity is needed. Developing a web-based psychotherapy tool with predesigned therapy modules and care processes can drastically increase care capacity for an overwhelmed mental health care system without sacrificing the quality of care. In this study, we aim to evaluate the feasibility and efficacy of this method of care delivery. By evaluating both the efficacy (through validated symptomology questionnaires) and feasibility (using focus groups), we can learn more about the real-world usefulness of e-CBT. Many of the current options for e-CBT are offered in nonsecure, nonscalable formats (email) or less effective (self-help) modalities, and by using this web-based platform, we can fill the gaps in the literature surrounding a large-scale psychotherapy clinic format for e-CBT delivery. Through the focus group findings, we can continue to better understand the accessibility issues associated with the remote delivery of mental health treatments and use these findings to develop more accessible options for patients. If proven feasible and efficacious, a web-based psychotherapy clinic can provide significant financial savings to the health care system through efficient use of clinician time, while providing an equitable and accessible method of treatment delivery for patients.

### Conclusions

The outcomes of this study will be shared as a preprint through bioRxiv for the rapid dissemination of the findings. We will also hold multiple web-based workshops for other clinicians interested in implementing this approach and provide technical and academic support to deploy this solution in their respective practices. This will ensure that the findings can be efficiently incorporated into clinical practice across the country. This protocol and future publications or findings will be reported using the guidance for reporting interventional development studies in health research framework and the template for intervention description and replication [36,37].

## Appendix

Multimedia Appendix 1 Sample electronically delivered cognitive behavioral therapy module session.

## Abbreviations

CBT: cognitive behavioral therapy
e-CBT: electronically delivered cognitive behavioral therapy
HSREB: Health Sciences and Affiliated Teaching Hospitals Research Ethics Board
MDD: major depressive disorder
OPTT: Online Psychotherapy Tool
PHQ-9: Patient Health Questionnaire-9
